# Establishing machine learning models to predict the early risk of gastric cancer based on lifestyle factors

**DOI:** 10.1186/s12876-022-02626-x

**Published:** 2023-01-10

**Authors:** Mohammad Reza Afrash, Mohsen Shafiee, Hadi Kazemi-Arpanahi

**Affiliations:** 1 Department of Artificial Intelligence, Smart University of Medical Sciences, Tehran, Iran; 2 Department of Nursing, Abadan University of Medical Sciences, Abadan, Iran; 3 Department of Health Information Technology, Abadan University of Medical Sciences, Abadan, Iran

**Keywords:** Machine learning, Gastric cancer, Behavioral lifestyle, Prevention, Prognosis

## Abstract

**Background:**

Gastric cancer is one of the leading causes of death worldwide. Screening for gastric cancer greatly relies on endoscopy and pathology biopsy, which are invasive and pose financial burdens. Thus, the prevention of the disease by modifying lifestyle-related behaviors and dietary habits or even the prevention of risk factor formation is of great importance. This study aimed to construct an inexpensive, non-invasive, fast, and high-precision diagnostic model using six machine learning (ML) algorithms to classify patients at high or low risk of developing gastric cancer by analyzing individual lifestyle factors.

**Methods:**

This retrospective study used the data of 2029 individuals from the gastric cancer database of Ayatollah Taleghani Hospital in Abadan City, Iran. The data were randomly separated into training and test sets (ratio 0.7:0.3). Six  ML methods, including multilayer perceptron (MLP), support vector machine (SVM) (linear kernel), SVM (RBF kernel), k-nearest neighbors (KNN) (K = 1, 3, 7, 9), random forest (RF), and eXtreme Gradient Boosting (XGBoost), were trained to construct prognostic models before and after performing the relief feature selection method. Finally, to evaluate the models’ performance, the metrics derived from the confusion matrix were calculated via a test split and cross-validation.

**Results:**

This study found 11 important influence factors for the risk of gastric cancer, such as Helicobacter pylori infection, high salt intake, and chronic atrophic gastritis, among other factors. Comparisons indicated that the XGBoost had the best performance for the risk prediction of gastric cancer.

**Conclusions:**

The results suggest that based on simple baseline patient data, the ML techniques have the potential to start the prescreening of gastric cancer and identify high-risk individuals who should proceed with invasive examinations. Our model could also considerably lessen the number of cases that need endoscopic surveillance. Future studies are required to validate the efficacy of the models in a larger and multicenter population.

## Introduction

Gastric cancer (also known as stomach cancer) is the fourth most prevalent neoplastic disease and the second leading cause of cancer-related deaths worldwide [1]. Gastric cancer, with a yearly incidence of about 7300 individuals, is one of the five most prevalent malignancies in the Iranian population. This disease is the first cause of cancer-related deaths in both sexes in Iran because the majority of patients are diagnosed in the advanced stages of the disease. Moreover, the 5-year survival rate in Iran is estimated at less than 25% [2]. A large proportion of patients with gastric cancer typically have no specific symptoms, and some of the early signs in patients are similar to gastritis or indigestion; therefore, gastric cancer is easily disregarded by patients. By the time their symptoms are noticeable, most of the patients have developed advanced gastric cancer. As a result, cancer invades adjacent tissues, and in such cases, treatments are ineffective and challenging, and the patient dies in a short while. The 5-year chance of surviving gastric cancer in a patient diagnosed in the early stages is more than 80%, which is significantly higher than the survival rate of a patient diagnosed in the advanced stages [3–5], highlighting the urgent need for an early screening method to improve the detection of gastric cancer. Individuals with low-risk gastric cancer should also be monitored to minimize the likelihood of advancing to high-risk stages. Therefore, preventing risk factors that contribute to the formation and development of gastric cancer should be a priority in healthcare system programs [6].

Endoscopy along with pathology biopsy is the current gold standard in the screening and detection of gastric cancer [7]. However, some patients, especially in rural and remote areas, avoid endoscopy or surgery due to the invasive nature and cost of this procedure [8]. If an individual is predicted to have a high risk of developing gastric cancer, preventative measures can be taken in advance. On the other hand, if the prognosis indicates that the patient has a low risk of developing gastric cancer, endoscopic examinations of the upper gastrointestinal tract, which are associated with possible risks and high screening costs, can be avoided or minimized. A large-scale survey of 200,000 individuals who underwent endoscopic examinations revealed a side effect rate of 0.13% and a mortality rate of 0.004%. Therefore, endoscopic screening for gastric cancer has been suggested in several subgroups of patients at risk [9, 10]. A meta-analysis study conducted in 2018 showed that population-based endoscopic screening in Asian countries significantly reduces the risk of death from gastric cancer. However, establishing a population-based endoscopic screening program in clinical practice is neither cost-effective nor practical [11]. Hence, the adoption of non-invasive techniques or models for the diagnosis of gastric cancer is of great importance.

Thus far, no non-invasive measures have been taken to diagnose gastric cancer with high sensitivity and specificity. Early diagnosis of gastric cancer and the subsequent early treatment are crucial to improve survival and reduce mortality from this cancer. Therefore, due to the complexity and interlinking factors that are causally related to gastric cancer, it is increasingly urgent to adopt non-invasive and time-saving diagnostic methods with high accuracy to minimize imprecision and uncertainty in the diagnosis of gastric cancer.

The adoption of artificial intelligence (AI)-based solutions, such as machine learning (ML), can overcome the restrictions of invasive diagnostic procedures in the screening and diagnosis of gastric cancer due to their computational capacity. ML techniques are well-known tools for developing predictive and data analysis models and can implicitly extract useful information from raw datasets [12]. ML can extract hidden relationships and patterns from large and high-dimensional data in single- or multicenter datasets [13, 14]. ML models are automatically created based on training data that can be used to make inferences or decisions in uncertain conditions without being explicit programming [15]. By capturing multifaceted nonlinear relations in the datasets, ML algorithms can increase the prediction accuracy more than traditional statistics techniques [16, 17].

Many studies have used ML techniques to predict gastric cancer up until now. Liu et al. used ML to predict gastric cancer with an accuracy of 77% [18]. Cai et al. performed univariate and multivariate analyses for gastric cancer prediction using demographic, dietary, and medical history as input data [19]. Safdari et al. developed a system for earlier diagnosis of gastric cancer using fuzzy logic with a sensitivity of 92.1% and a specificity of 83.1% [20]. Su Y et al. detected gastric cancer using a decision tree (DT) classification of mass spectral data with an accuracy of 86.4% [21]. Brindha et al. utilized dietary and lifestyle features to predict etiological factors of early gastric cancer and trained several supervised ML algorithms, including naive Bayes, logistic regression (LR), and multilayer perceptron (MLP). Their results showed that naive Bayes has the best performance with an accuracy of 90% as compared to the other models [22]. Mortezagholi et al. employed endoscopy images as attributes for a case–control study using ML techniques, including the support vector machine (SVM), DT, naive Bayesian model, and k nearest neighborhood (KNN) to predict patients with gastric cancer [23]. They reported that SVM with an accuracy of 90.8% exhibits the best performance compared to the other models [8]. Taninaga et al. showed that eXtreme Gradient Boosting (XGBoost) outperformed LR in predicting gastric cancer using comprehensive longitudinal data with the highest area under the curve (AUC) value (0.899) [10]. Several studies have applied neural network methods to detect gastric cancer based on endoscopic images with high sensitivity [24, 25]. In the study of gastric cancer, ML techniques are mainly used to analyze endoscopic images, which are obtained through invasive methods [24, 26]. In contrast, analyzing lifestyle-related factors is non-invasive and inexpensive. Gastric cancer is largely reliant on lifestyle-related factors and can be prevented with a change of diet and habits [27]. Therefore, in this study, we aimed to predict gastric cancer based on lifestyle and historical data using ML methods. Thus far, numerous ML methods have been developed in the medical field. Each of these ML methods has a different algorithm and nature of work. If chosen appropriately, all these models will perform at their peak [28]. The selection of ML models is dependent on the data (the type and specific characteristics of each dataset, such as structured or unstructured, number of dimensions, number of samples, and other similar factors) as well as the desired performance [29]. In the current study, we chose six ML algorithms that performed well on structured and unstructured datasets and on datasets with several dimensions (SVM, KNN, random forest (RF)), an ML model that could solve complex nonlinear problems randomly (MLP), and a model that has the ability to set multiple hyperparameters to achieve high accuracy (XGBoost) [30–33].

The structure of the manuscript is as follows: First, the dataset is presented. The ML techniques used in this paper are then described in detail. After that, the results of comparing ML techniques are shown. In the next stage, the most accurate model for predicting gastric cancer based on the results of performance evaluation metrics is reported.

## Methods

### Study design and experiment environment

**Fig. 1:**
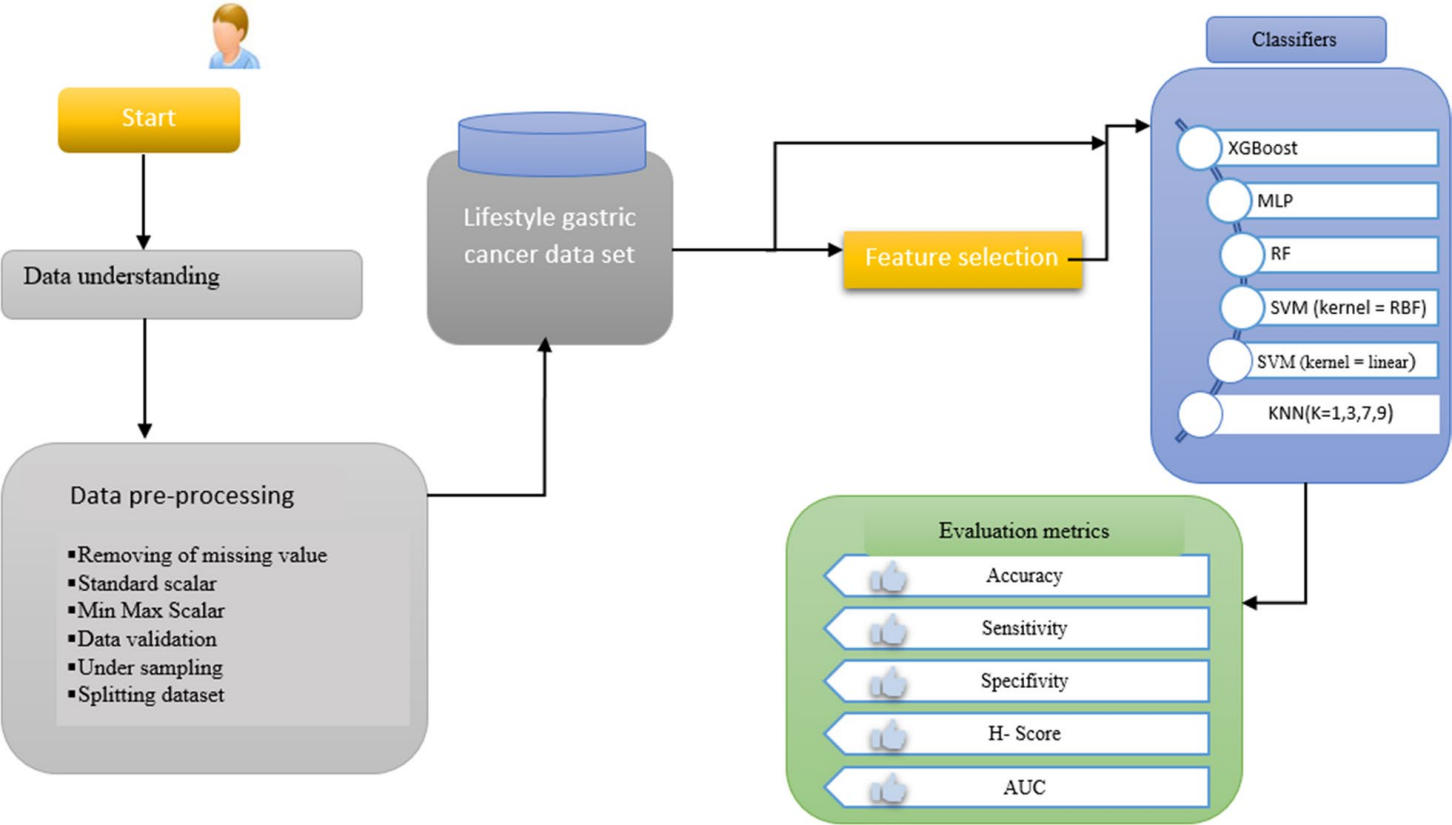
Block diagram of the proposed system for gastric cancer risk prediction

This is a retrospective, single-center study that was conducted in 2022. A dataset was collected from the Ayatollah Taleghani database affiliated with Abadan University of Medical Sciences, Abadan City, Iran. Six ML-based models were developed for the prediction of gastric cancer using lifestyle-related factors. This study was conducted based on the cross-industry standard process for data mining (CRISP-DM). The prediction models were developed using Python programming language in five main CRISP stages including data understanding, pre-processing, feature selection, model training, and evaluation, as shown in the block diagram below (Fig. 1).

### Dataset description and participants

The dataset used in this study was obtained from the gastric cases referred to the internal clinic of Ayatollah Taleghani Hospital in Abadan, Iran, during 2015–2021. The patients’ information was reviewed and extracted by a health information management expert. The patients who were referred to the clinic for the screening, diagnosis, and treatment of gastric cancer were included in this study. A total of 2029 individuals (429 patients vs. 1780 healthy controls) participated in the study. The analyzed dataset contained descriptive information about the respondents (28 features) and the outcome of the gastric cancer risk (one feature), which can be viewed as the dependent variable (Table 1).

**Table 1:** Gastric cancer variables

Variable name	Values
Age (year)	18–94
Gender	Male, female
BMI (kg/m^2^)	< 25, > > 25
Blood type A	Yes/No
Marital status	Single, married
Family history of cancer	Yes/No
Depression	Yes/No
Stress status	NO–mild–sever
Income level	Lowest–middle–highest
Education level	Uneducated, high school, university
Residence status	Rural, town
High salt intake	Yes/No
High fat foods status	Yes/No
Alcohol consumption	Yes/No
Smoking	Yes/No
Physical activity	Yes/No
Fruits intake	Low–middle–high
Red meat consumption	Low–middle–high
Gastric cancer screening	Participated-did not participate
Weight loss	Yes/No
Helicobacter pylori test	Negative, positive
Upper abdominal fullness	Yes/No
Abdominal pain	Yes/No
Stomach polyp	Yes/No
Recurrent nausea and vomiting	Yes/No
Stomach or duodenal ulcers	Yes/No
Previous stomach surgery	Yes/No
Chronic atrophic gastritis	Yes/No

### Preprocessing the dataset

Data preprocessing is an essential step in the CRISP-DM method, and it has a significant impact on the performance of data mining techniques. The objectives of data preprocessing are to cleanse the outlier, remove the noisy data, impute missing values, and convert the data into a suitable format for more reliable and accurate data analysis. In the first phase of preprocessing steps, we employed the interquartile range rule to detect outliers and normalize the dataset by using the min–max method. To impute missing values, mean and regression-based methods were applied. We also deleted the rows with more than 70% missing values. The Z-score standardization method was used as a data distribution-based data scaling, and for data range-based scaling, the min–max method was employed. In the preprocessing phase, 240 records of the dataset were deleted, and after removing these records, the number of the cases of the dataset was reduced to 2029 records. Since group distribution in the dataset used for this study is imbalanced, one of the groups contains 1780 samples (healthy individuals) while the other has 429 samples (patients). Therefore, we created a new dataset by approximating the group with fewer samples (patient group = 429) to the group with a larger number of samples (healthy group = 1780 individuals), such that the samples of the group with fewer cases in the dataset are randomly reproduced. In this study, the **s**ynthetic minority oversampling technique **(**SMOTE) was applied to the resampling process. The SMOTE method is the most common and effective oversampling method, which is applied in various fields to balance the datasets [34, 35].

### Feature selection

One of the fundamental issues with many data mining tasks is to determine and specify relationships between attributes in the dataset and outcome. Feature selection is one of the main phases of a successful data mining process, especially in problems with a large number of dimensions or variables in the dataset. Feature selection is defined as the process of determining relevant variables and removing irrelevant ones [36]. In the present study, the relief feature selection algorithm was implemented to reduce the number of features and combinations in order to obtain the most important predictors. Relief is a method for the random selection of relevant attributes based on variables' weight. This algorithm assigns weights to all the variables. The most important attributes to the outcome have higher weight values, whereas the other attributes have lower weights [37].

### Training and evaluation of ML classifiers

In order to develop an early prediction model for gastric cancer risk, a total of six ML algorithms were used: MLP, SVM (linear kernel), SVM (RBF kernel), KNN (K = 1, 3, 7, 9), RF, and XGBoost. To implement these models, we experimentally tuned the hyperparameters on the training split of the dataset based on the cross-validation (CV) method. The performance of the ML algorithms was evaluated using the holdout technique, a method for out-of-sample assessment where the dataset was split into two parts (70% training and 30% test). The ML models were then trained on the first split of the dataset and tested on the other part. In addition, the K-fold CV method (K = 10) was applied to evaluate the performance of the best algorithms to overcome a feasible biased error estimate. Five performance evaluation metrics were selected and reported for each ML technique to compare the performance of classifiers, which is common in medical prediction studies. The performance evaluation metrics of the classifiers are listed below, along with their definitions:

1 $$classification \;accuracy = \frac{TP + TN}{{TP + TN + FP + FN}}*100$$

2 $$classification \;sensitivity = \frac{Tp}{{TP + FN}}*100$$

3 $$classification \;specificity = \frac{TN}{{TN + FP}}*100$$

4 $$classification \;error = \frac{FP + FN}{{TP + TN + FP + FN}}*100$$

### Ethical consideration

This study was approved by the ethics committee board of Abadan University of Medical Sciences (Ethics code: IR.ABADANUMS.REC.1401.013). To protect the privacy and confidentiality of patients, we concealed the unique identification information of all patients in the process of data collection and presentation. The present study adhered to the principles expressed in the Declaration of Helsinki.

## Results

### Characteristics of patients

After applying the exclusion criteria and conducting a quantitative analysis of patients’ records, 2029 patients were found to be eligible. Of the 2029 participants in the study, 1094 (54%) were male and 1015 (46%) were female, and the age of the participants was between 18 and 94. In total, 1780 (88%) of the study subjects were healthy controls, and 429 (22%) were patients. The descriptive statistics for the 2029 samples in this dataset are shown in Table 2.

**Table 2:** **The descriptive statistics of variables**

Variable name		Total	With gastric cancer (429)	No gastric cancer(1780)
			N	N
Age	>>40	505	23	482
	40-65	937	147	790
	65>>	767	259	508
Gender	Male	1025	192	833
	Female	1184	237	947
BMI (kg/m2)	>25	1660	194	1466
	<<25	549	235	314
Blood type A	Yes	402	95	307
	No	1807	334	1473
Marital status	Single	744	90	654
	Married	1465	339	1126
Family history of cancer	Yes	167	14	153
	No	2042	415	1627
Depression status	Yes	525	194	331
	No	1684	235	1449
Stress status	NO	626	36	590
	Mild	1293	190	1103
	Sever	290	203	87
Income level	Lowest	1074	176	898
	Middle	811	214	597
	High	324	39	285
Education level	Uneducated	360	47	313
	high school	681	192	489
	University	1168	190	978
Residence status	Rural	656	84	572
	Town	1553	345	1208
High salt intake	Yes	1161	162	999
	No	1048	267	781
High fat foods status	Yes	1236	244	992
	No	973	185	788
Alcohol consumption	Yes	140	28	112
	No	2069	401	1668
Smoking	Yes	464	74	390
	No	1745	355	1390
Physical activity	Yes	651	52	599
	No	1558	377	1181
Fruits consumption	Low	416	114	302
	Middle	1473	284	1189
	High	320	31	289
Red meat consumption	low	1377	86	1291
	Middle	577	195	382
	High	255	148	107
Gastric cancer screening	Yes	370	14	356
	No	1839	415	1424
Weight loss	Yes	378	186	192
	No	1831	243	1588
Helicobacter pylori infection	Yes	307	174	133
	No	1902	255	1647
Upper abdominal fullness	Yes	501	286	215
	No	1708	143	1565
Abdominal pain	Yes	600	236	364
	No	1609	193	1416
Stomach polyp	Yes	210	80	130
	No	1999	349	1650
Recurrent nausea and vomiting	Yes	330	149	181
	No	1879	280	1599
Stomach or duodenal ulcers	Yes	322	217	105
	No	1887	212	1675
Previous stomach surgery	Yes	237	69	168
	No	1972	360	1612
Chronic atrophic gastritis	Yes	260	112	148
	No	1949	317	1632

### The results of selected features using the relief feature selection algorithm

A total of 11 features were selected due to their positive correlation with gastric cancer by performing the Relief feature selection algorithm. These features are Helicobacter pylori infection, high salt intake, chronic atrophic gastritis, consumption of fruits, stomach or duodenal ulcers, weight loss, consumption of high-fat foods, educational level, smoking, stress status, and weight. The key features selected by the Relief feature selection algorithm and their scores are presented in Table 3.

**Table 3:** Variables selected by feature selection

Order	Feature name	Score
1	Helicobacter pylori infection	4.10
2	High salt intake	1.807
3	Chronic atrophic gastritis	1.802
4	Fruits consumption	1.59
5	Stomach or duodenal ulcers	1.45
6	Weight loss	1.42
7	High fat foods	1.36
8	Education level	1.34
9	Smoking	1.22
10	Stress status	1.19
11	Weight loss	1.14

**Fig. 2:**
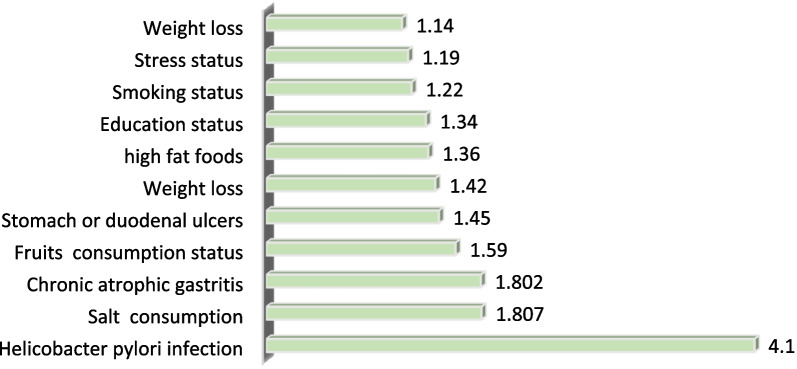
The most important features selected by the Relief feature selection algorithm

Based on the results of the Relief feature selection algorithm (Table 3), Helicobacter pylori infection is three times more effective than the other attributes for the early prediction of gastric cancer. High salt intake, chronic atrophic gastritis, and low consumption of fruits are significantly associated with a high risk of gastric cancer. In contrast, weight loss and stress were ranked as the 10th and 11th most important risk factors in the prediction of gastric cancer, respectively (see Fig. 2).

### The results of the tuning of hyperparameters

In order to improve the performance of ML techniques, many methods can be used to tune hyperparameters. In this study, the randomized search CV method was employed for the tuning of parameters and the optimization of ML techniques. Table 4 indicates the best hyperparameter classifier for the early prediction of gastric cancer based on lifestyle factors.

**Table 4:** Hyperparameters selected to be fed into the classifiers for the early prediction of gastric cancer

Num	ML models	Hyper-parameters	F-score
1	RF	(‘verbose’:2,’random_state’:888,’n_estimators’:10,’max_deph’:9,’criterion’: gini’)	85.31
2	MLP	‘Learning rate’ = ’constant’, hidden_layer_size’ = (80, 80, 80), ‘alpha’ = 0.08, ‘activation’ = ’rulo’	87.6
3	SVM (kernel = linear)	C = 100, G = 0.0001	83.04
4	SVM (kernel = RBF)	C = 10, G = 0.001	81.9
5	XGBoost	‘min_chid_weigh’ = 1’max_depht’ = 14,’learning_rate’ = 0.2, ‘gamma’ = 0.4, ‘colsample_bytree’ = 0.5	81.02

### The results of k-fold CV for classifiers’ performance on full features and selected features

In the present study, full features and features selected by the Relief feature selection algorithm were tested on six classification algorithms using the 10-fold CV methods. In the tenfold CV method, 90% of the dataset was used for training the algorithms and only 10% was tested. The mean metrics of tenfold methods were measured. Additionally, different metrics values were passed through classification algorithms. At first, we trained and tested the data mining algorithms with all the dataset features. Then we fed 11 features into the selected classifiers. To better represent the performance of classifiers, some figures were created for classification accuracy, sensitivity, specificity, AUC, and the H-score metric. The performance of the ML algorithms on all features and selected features using the tenfold CV method is displayed in Table 5.

**Table 5:** The results of tenfold CV for models’ performance metrics on full features and selected features

	SVM (RBF)		XGBoost		SVM (Linear)		RF		MLP	
	Full features	Selected features	Full features	Selected features	Full features	Selected features	Full features	Selected features	Full features	Selected features
Accuracy	0.624	0.817	0.652	0.8341	0.593	0.7807	0.622	0.814	0.590	0.7016
Specificity	0.619	0.7985	0.637	0.859	0.578	0.731	0.594	0.7733	0.591	0.7182
Sensitivity	0.611	0.8024	0.668	0.837	0.573	0.8117	0.627	0.801	0.586	0.708
AUC	0.584	0.7995	0.651	0.849	0.584	0.792	0.631	0.798	0.572	0.7051
H-Score	0.617	0.7925	0.653	0.832	0.580	0.7817	0.6287	0.7931	0.594	0.712

**Fig. 3:**
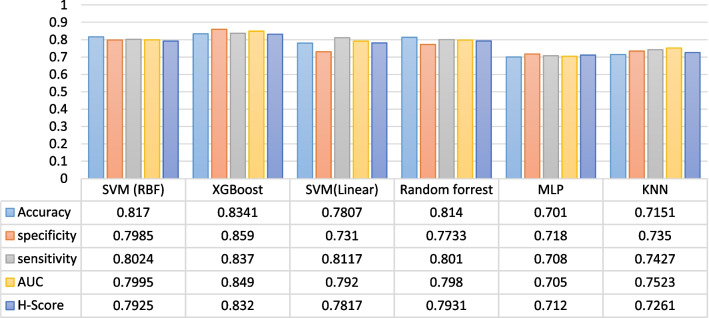
Comparison of the performance of the six best ML models on the selected features

According to the results presented in Table 5, the performance of classifiers on the selected features was better than on full features. When the selected factors were included in the model, the results show that the XGBoost classifier yielded an accuracy of 83.4%, a sensitivity of 83.7%, a specificity of 85.9%, an AUC of 84.9%, and an H-score of 83.2%. While the KNN classifier for K = 7 obtained the mean accuracy of 67.98%, the mean sensitivity of 66%, a specificity of 70.2%, an AUC of 66.8%, and a mean H-score of 69.14% when all features were fed into the classifiers (Table 6). The results of the six best experiments of classifiers on the selected features are shown in Fig. 3.

**Table 6:** The results of tenfold CV for models’ performance metrics on full features and selected features

KNN										
K	Accuracy		Specificity		Sensitivity		AUC		H-Score	
	Full features	Selected features	Full features	Selected features	Full features	Selected features	Full features	Selected features	Full features	Selected features
1	0.664	0.7126	0.6737	0.7882	0.6587	0.733	0.6538	0.710	0.6481	0.6910
3	0.6538	0.7223	0.6819	0.7647	0.6597	0.7255	0.6767	0.7365	0.6837	0.7153
7	0.6798	0.7151	0.7022	0.735	0.6696	0.7427	0.6684	0.7523	0.6914	0.7261
9	0.6524	0.7369	0.6831	0.7813	0.661	0.7407	0.6944	0.7312	0.602	0.7231

**Fig. 4:**
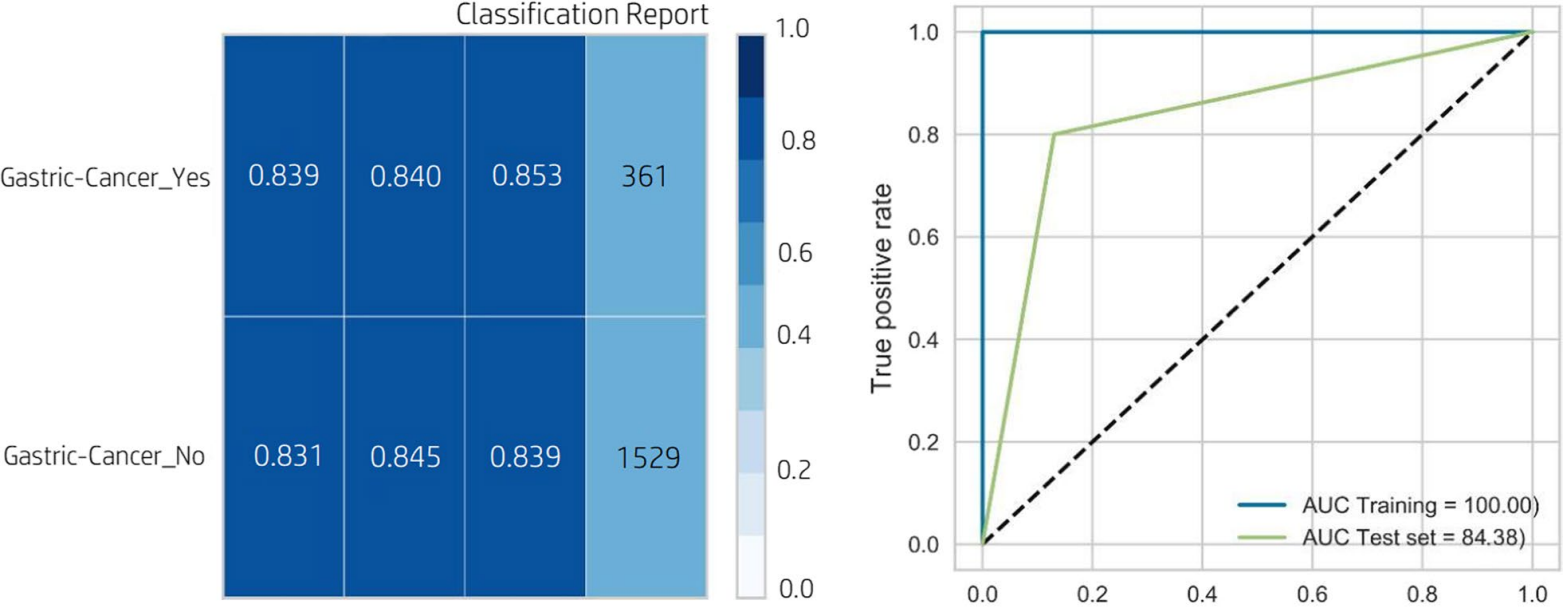
The classification report and ROC curve for the XGBoost classifier

According to Fig. 3, the performance of the XGBoost classifier was better than that of the five other algorithms (a mean accuracy of 83.7%, a mean specificity of 85.9%, an average sensitivity of 83.7%, an AUC of 84.9, and an H-score of 83.2). In Fig. 3, the worst performance was observed for the MLP classifier with an accuracy, specificity, sensitivity, AUC, and H-score of 70.1%, 71.8%, 70.5%, 70.8%, and 71.2%, respectively. The AUC and classification report of the XGBoost classifier, which was selected as the best classifier in the prediction of gastric cancer, are shown in Fig. 4.

## Discussion

It is important to provide accurate and rapid screening for gastric cancer since this malignancy is very treatable when diagnosed in early phases. Therefore, implementing procedures for the screening and diagnosis of gastric cancer is highly beneficial. In this regard, a timely and reliable screening method enables rapid detection of the disease, and the subsequent timely interventions can help boost the likelihood of patient survival (2, 10). In this study, we developed non-invasive and cost-effective predictive models using six ML algorithms for gastric cancer risk assessment to distinguish high-risk patients with gastric cancer from the general population. Due to the fact that gastric cancer has multiple features with many potentially important confounders, it is a great challenge for clinicians to consider and analyze all the engaging features and decide on the patient's condition. It also raises the likelihood of a physician error during the decision-making process of disease diagnosis [38]. Thus, it is desirable to use an intelligent method that has the ability to learn the problem and generalize it to other situations. In this study, six methods of MLP, SVM (linear kernel, RBF kernel), KNN, RF, and XGBoost were proposed to classify gastric cancer patients. Based on the results, the XGBoost had the best performance in predicting gastric cancer risk in comparison with the other ML techniques using the stated evaluation metrics.

Several studies have been conducted with the aim of improving the early screening accuracy of gastric cancer through the use of ML. The predictive models in previous studies were developed based on diagnostic, laboratory, pathology, and imaging data and were not related to lifestyle data and individual habits and behaviors. Therefore, the advantage that distinguishes our study was that we obtained relatively significant results by applying ML methods based on data on the lifestyle features and behavior of individuals. Moreover, our study used feature selection to select the most significant lifestyle-based variables in order to maximize the capability of the models when compared to the analysis of all variables in the dataset. Feature selection enhances the accuracy, specificity, and sensitivity of the classifiers and decreases the running time of the predictive system. In this study, we used the ML algorithms due to the fact that these methods resulted in better predictions than the conventional statistical techniques when dealing with a large number of features with complicated relationships [39, 40]. Although statistical models can easily determine the relationship between dependent and independent variables, they cannot handle a large amount of variables with different types and intricate associations [41, 42]. If the aim of the study is to improve the performance of predictive models and the interpretation of models is of secondary importance, researchers prefer to develop ML models to achieve satisfactory predictions [40].

The main advantage of our study was that it estimated the risk of gastric cancer based on lifestyle-related factors. Some researchers have incessantly focused on medical equipment and detection reagents to improve the screening of gastric cancer, and the results of their studies were applied to clinical gastroscopy and biopsy [43, 44]. A few studies have combined genetic, proteomics, and molecular biology to detect gastric cancer [45–48]. However, owing to their limitations, such as invasiveness, intricacy, high cost, or low adaptability, the diagnostic methods have not been widely adopted in clinical practice for gastric cancer screening. Tumor markers, e.g., CEA, CA199, CA125, and CA724, are generally used for the diagnosis of gastric cancer. But, the sensitivity and accuracy of these non-invasive features are not satisfactory [49–51]. Contrary to the abovementioned studies, we applied ML techniques to stratify the risk of gastric cancer, which is a non-invasive approach. Patients were first examined by the optimal ML models developed, and then the high-risk cases were referred to specialized centers for further diagnostic procedures, such as endoscopy and pathology biopsy. The non-invasive gastric cancer screening approach developed in our study is highly adaptable and low-cost, which increases the coverage of gastric cancer screening in clinical practice.

Nevertheless, this study also had some limitations. First, the participants in the study included patients from a single care center, which limits the generalizability of the results to larger populations. Second, there was some subjective bias in selecting variables and examining lifestyle behavior that may affect the predictive results. Another limitation of this study was a retrospective analysis using registered data, which reduces the external validity of the results. Thus, future testing in a larger population is recommended.

## Conclusions

This study utilized the routine available non-invasive features to implement six models for screening the risk of gastric cancer. The XGBoost model demonstrated better performance than the other ML models and can be applied to assist clinicians in the screening of gastric cancer risk in accordance with the Iranian health referral system and hierarchical leveling of healthcare services, which will improve the early screening of gastric cancer on a large scale. The ML models may identify high-risk patients with gastric cancer early, which draws the attention of clinicians and patients, and appropriate and timely interventions will be implemented to improve the patients’ survival chance and quality of life. This study can also aid clinical researchers in choosing and implementing the optimal prediction models and evaluating the main influencing features.

## Data Availability

The datasets used and/or analyzed during the current study are available from the corresponding author on reasonable request.

## Abbreviations

MLP: Multilayer perceptron
SVM: Support vector machine
KNN: Nearest neighbors
XGBoost: EXtreme gradient boosting
ML: Machine learning
AI: Artificial intelligence
SMOTE: Synthetic minority oversampling technique
